# Investigation of an Outbreak of COVID-19 in a French Nursing Home With Most Residents Vaccinated

**DOI:** 10.1001/jamanetworkopen.2021.25294

**Published:** 2021-09-13

**Authors:** Catherine Burugorri-Pierre, Carmelo Lafuente-Lafuente, Christel Oasi, Emmanuel Lecorche, Sylvie Pariel, Cristiano Donadio, Joël Belmin

**Affiliations:** 1 Etablissement d’Hébergement pour Personnes Agées Dépendantes Léon Dubédat, Biscarrosse, France; 2 Service de Gériatrie, Hôpital Charles Foix, Assistance Publique–Hopitaux de Paris, Ivry-sur-Seine, France; 3 Faculté de Médecine Sorbonne, Sorbonne Université, Paris, France; 4 Clinical Epidemiology and Ageing Unit, Institut National de la Santé et de la Recherche Médicale U955, Institut Mondor de Recherche Biomédicale, Créteil, France; 5 Laboratoire Cerba, Saint-Ouen L’Aumône, France

## Abstract

This cohort study investigates an outbreak of COVID-19 among residents and health care professionals in a French nursing home in which most residents were fully vaccinated against COVID-19.

## Introduction

There is great hope that vaccination against SARS-CoV-2 will decrease the burden of COVID-19 on nursing home (NH) residents, who have been significantly affected by this pandemic. The efficacy and effectiveness of COVID-19 vaccines are not well known in this population. Recent immunogenic studies have found decreased titers of postvaccine neutralizing antibodies against SARS-CoV-2 among NH residents, suggesting the potential for diminished effectiveness in this population.^1,2^ We report an outbreak of COVID-19 in a French nursing home where most residents had been fully vaccinated with the BNT162b2 vaccine.

## Methods

This cohort study was conducted according to the principle of the Declaration of Helsinki. The president of the Comité de Protection des Personnes Ile-de-France VI determined that this study was exempt from ethics committee review and oral or written participant consent according to the French law (décret No. 2016-1537, November 17, 2016). Residents of the facility and their families were informed that a report about the outbreak would be published using deidentified data. This report follows the Strengthening the Reporting of Observational Studies in Epidemiology (STROBE) reporting guideline for cohort studies.

The study reports an outbreak that occurred in a 77-bed NH located in Biscarrosse, France, in which a vaccine campaign was conducted using the BNT162b2 vaccine in January and February 2021. Several weeks after this campaign, a resident contracted COVID-19 outside of the facility. To control the spread of the infection, all residents and health care professionals who accepted the procedure repeatedly underwent nasal swabbing for reverse transcriptase–polymerase chain reaction (RT-PCR) testing for SARS-CoV-2. An outbreak of COVID-19 ensued, nonetheless. We followed up all individuals diagnosed with COVID-19 and extracted clinical data from residents’ medical records. Information about health care professionals was obtained from administrative records kept by the NH director. Additional methods are detailed in eMethods in the Supplement. These patients and data have not been previously reported. Data were analyzed from March 19 through April 18, 2021.

## Results

The index infection occurred in a resident who came into contact with a visitor who had symptoms. The visitor was diagnosed with COVID-19 and informed the facility of this diagnosis 4 days later. The resident was at that time asymptomatic with a positive RT-PCR test result, and she was isolated. She became symptomatic 2 days later and was treated with nasal oxygenation, fluid infusion, and anticoagulants.

The results of RT-PCR tests are presented in the Table. Among 74 residents (16 men [22.2%]; mean [SD] age, 87.8 [7.5] years), 72 individuals (97.3%) were vaccinated, including 70 residents who were fully vaccinated (ie, received 2 doses of BNT162b2 >14 days before the outbreak) and 2 residents who were partially vaccinated (ie, received 1 dose >14 days before the outbreak). In total, 17 residents (23.0%) were diagnosed with COVID-19 (5 men [29.4%] and 12 women [70.6%]; mean [SD] age, 87.6 [8.9] years). The individuals diagnosed with COVID-19 included 1 unvaccinated resident, 2 partially vaccinated residents, and 14 fully vaccinated residents. Among individuals with diagnoses, 8 residents developed severe disease, 2 were hospitalized, and 1 individual (the unvaccinated resident) died (Figure).

**Table.** RT-PCR Test Results, Confirmed Diagnoses, and Deaths by Vaccination Status

Outcome	Residents (n = 74)		Health care professionals (n = 102)	
	Vaccinated (n = 72)	Unvaccinated (n = 2)	Vaccinated (n = 34)	Unvaccinated (n = 68)
RT-PCR positive test result, No./No. total tests^a^				
Day 3	3/70	1/2	0/32	0/65
Day 7 or 8	10/59	0/1	1/34	4/68
Day 15	2/52	0/1	2/32	6/65
Confirmed COVID-19 diagnosis, No. (%)				
Any	16 (22.2)	1 (50.0)	3 (8.8)	9 (13.2)
Symptomatic COVID-19, No. (%)	14 (87.5)	1 (100)	0	5 (55.6)
Severe COVID-19, No. (%)	7 (50.0)	1 (100)	0	0
COVID-19–related death, No. (%)	0	1 (100)	0	0

Abbreviation: RT-PCR, reverse transcriptase–polymerase chain reaction.

^a^ RT-PCR results are for tests performed after the index diagnosis (ie, day 0). Positive results are out of the total number of individuals receiving RT-PCR tests that day.

**Figure.**
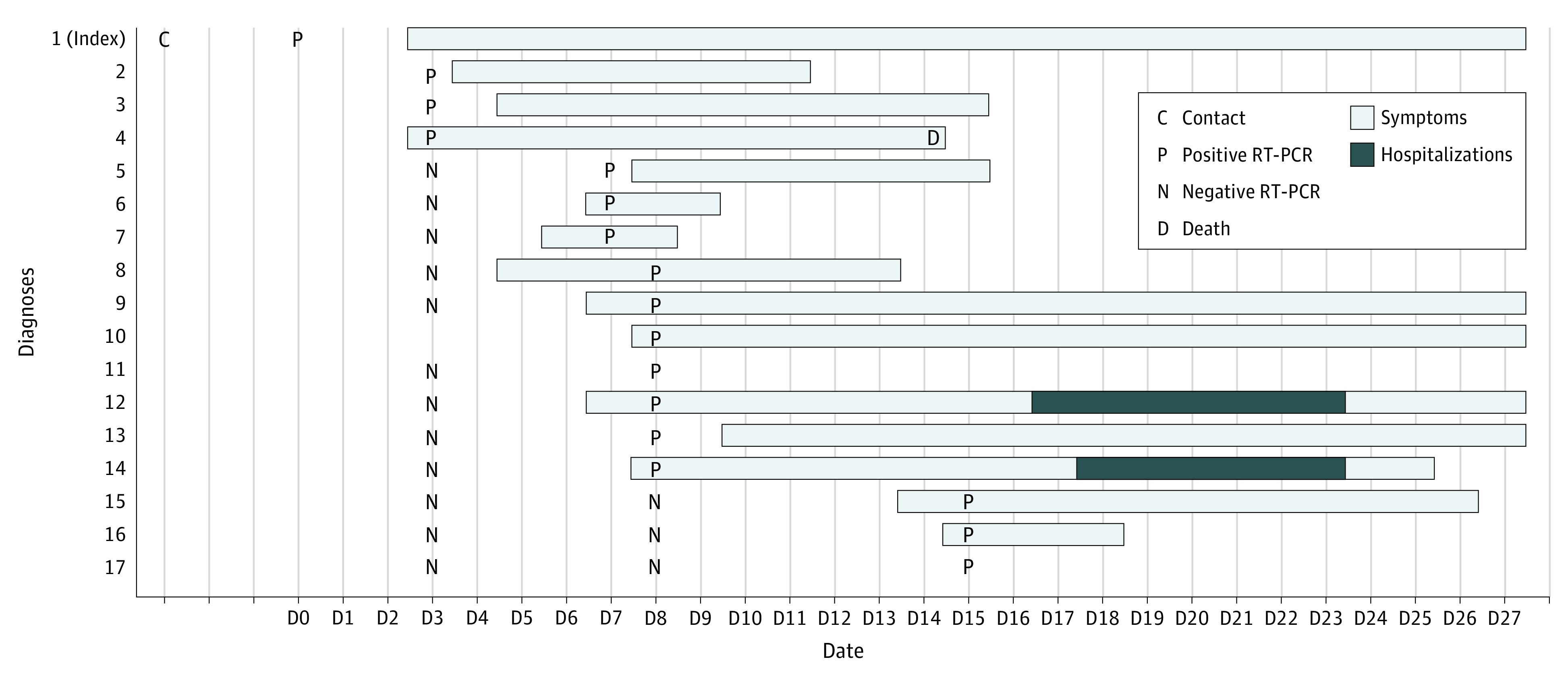
Timeline of Test Results, Symptoms, Symptom Duration, and Complications Outcomes are among 17 nursing home residents with a confirmed diagnosis of COVID-19. Individual No. 4 was unvaccinated, and all others were vaccinated with BNT162b2 vaccine; individuals No. 7 and 17 had only 1 dose. RT-PCR indicates reverse transcriptase–polymerase chain reaction.

Among 102 health care professionals, 34 individuals were vaccinated (33.3%), 68 individuals were unvaccinated (66.7%), and 12 individuals were diagnosed with COVID-19 (11.8%). Of these, 5 individuals became symptomatic and none had severe disease. Among health care professionals who were unvaccinated, 9 individuals (13.2%) were diagnosed with COVID-19, and among health care professionals who were previously vaccinated, 3 individuals (8.8%) were diagnosed (including 1 individual fully vaccinated with the BNT162b2 vaccine and 2 individuals partially vaccinated with the ChAdOx1 nCoV-19 vaccine).

The SARS-CoV-2 variant B.1.1.7 was identified among all residents and health care professionals who were diagnosed with COVID-19. Viral genomes were sequenced for 9 samples obtained from 4 residents and 5 health care professionals. The profile of this variant revealed no specific mutations in the spike gene.

## Discussion

This cohort study’s findings suggest that an outbreak of COVID-19 can occur among fully vaccinated NH residents. The study found evidence of transmission among vaccinated residents, but few individuals who were infected developed severe disease and 1 patient, who was unvaccinated, died. Moreover, these outcomes occurred in a setting in which approximately 30% of staff members were vaccinated.

To our knowledge, this is the first outbreak due to the B.1.1.7 COVID-19 variant described among individuals well vaccinated against SARS-CoV-2. Cavanaugh et al^3^ reported a COVID-19 outbreak associated with a SARS-CoV-2 R.1 lineage variant that occurred in a US skilled nursing facility where 90% of residents were fully vaccinated.

The occurrence of the outbreak described in our study despite vaccination may be associated with impaired immune function associated with immunosenescence and health conditions, such as malnutrition, diabetes, and cancer, that are frequent among NH residents.^4^ Several studies^1,2,4,5,6^ have found reduced antibody response to several vaccines, including BNT162b2, in this population.

Our study’s findings suggest that SARS-CoV-2 vaccination may not be sufficient as the sole means to prevent COVID-19 among NH residents and that other prevention measures should not be abandoned yet in these settings. More research is needed to improve the effectiveness of SARS-CoV-2 vaccines in this population.

## Notes

Supplement.
eMethods.
