# Supplementary material for: Investigation of an Outbreak of COVID-19 in a French Nursing Home With Most Residents Vaccinated
Source: JAMA Netw Open. 2021 Sep 13;4(9):e2125294. doi: 10.1001/jamanetworkopen.2021.25294 (PMC8438595; doi:10.1001/jamanetworkopen.2021.25294)
Supplement: Supplement. — eMethods. [file jamanetwopen-e2125294-s001.pdf]

Supplemental Online Content

Burugorri-Pierre C, Lafuente-Lafuente C, Oasi C, et al. Investigation of an outbreak of COVID-19 in a French nursing home with most residents vaccinated. *JAMA Netw Open*. 2021;4(9):e2125294. doi:10.1001/jamanetworkopen.2021.25294

eMethods.

This supplemental material has been provided by the authors to give readers additional information about their work.

© 2021 Burugorri-Pierre C et al. *JAMA Network Open.*

eMethods.

Case definition and virologic aspects

Confirmed cases of COVID-19 were diagnosed on the basis of a positive reverse transcriptase polymerase chain reaction (RT-PCR) test. SARS-CoV-2 was search by RT-PCR on nasal swabbing using Allplex SARS-CoV-2 assay (Seegene, Korea). The variant B.1.1.7 was detected by searching del69-70 and N501Y mutations using the ID- SARS-CoV-2/UK/SA Variant Triplex reactant (ID Solutions, France). Cases were considered symptomatic if one or more of the following symptoms or signs were present: fever, cough, difficult breathing, anosmia, diarrhea, pneumonia confirmed by chest imaging. SpO2 Cases were considered severe if oxygen saturation was <94% on room, respiratory rate >30 breaths/min, or lung infiltrates >50%, and/or if the disease required nasal oxygen, fluid infusion, or hospitalization. Next generation sequencing based on capture-enrichment approach was performed with Twist Respiratory Virus (103067; Twist Biosciences, San Francisco, CA) and Illumina sequencing. Bioinformatic analysis was performed using Nanoware pipeline, Life&Soft.

Prevention measures in the facility

Shortly before the outbreak, prevention relied primarily on SARS-CoV-2 vaccination for residents and health care professionals (HCPs), and hand hygiene, masking, and social distancing for HCPs. Resident visits were allowed. When the outbreak was diagnosed, additional prevention measures were implemented: screening residents and HCPs for asymptomatic COVID-19 by nasal swab, isolating positive residents, stopping work and expelling positive HCPs from the facility, and prohibiting resident visits.

© 2021 Burugorri-Pierre C et al. *JAMA Network Open.*
